# Assessment of genome stability in various breeds of cattle

**DOI:** 10.1371/journal.pone.0217799

**Published:** 2019-06-04

**Authors:** Ewa Wójcik, Małgorzata Szostek

**Affiliations:** Institute of Bioengineering and Animal Breeding, Siedlce University of Natural Sciences and Humanities, Siedlce, Poland; University of Illinois, UNITED STATES

## Abstract

Chromosomal instability is a type of genome instability involving changes in genetic information at the chromosomal level. The basic tests used to identify this form of instability are sister chromatid exchange (SCE) tests and identification of fragile sites (FS). SCE is the process by which sister chromatids become fragmented as a result of DNA strand breakage and reassembly, followed by exchange of these fragments. FS can be observed in the form of breaks, gaps or constrictions on chromosomes, which often result from multiple nucleotide repeats in DNA that are difficult to replicate. The research material was the peripheral blood of ten breeds of cattle raised in Poland, including four native breeds covered by a genetic resources conservation programme, i.e. Polish Red, Polish Red-and-White, White-Backed, and Polish Black-and-White, as well as Polish Holstein-Friesian, Simmental, Montbéliarde, Jersey, Limousine and Danish Red. Two tests were performed on chromosomes obtained from in vitro cultures: SCE and FS. The average frequency of SCE was 5.08 ± 1.31, while the incidence of FS was 3.45 ± 0.94. Differences in the incidence of SCE and FS were observed between breeds. The least damage was observed in the Polish Red and White-Backed breeds, and the most in Polish Holstein-Friesians. The most damage was observed in the interstitial part of the chromosomes. Age was shown to significantly affect the incidence of SCE and FS. Younger cows showed less damage than older ones (SCE: 4.84 ± 1.25; 5.34 ± 1.24; FS: 3.10 ± 0.88, 3.80 ± 0.92).

## Introduction

Chromosomal instability is one type of the genomic instability [1]. It involves changes in genetic information at the chromosome level [2]. These changes may cause disturbances in the functioning of the cell [3]. Basic cytogenetic diagnostic tests used to detect structural chromosomal instability include the sister chromatid exchange (SCE) test and identification of fragile sites (FS). Sister chromatid exchange is a natural phenomenon in the cell cycle [4,5]. SCE is a process involving the exchange of regions of parental strands in duplicated chromosomes between sister chromatids [6]. They are the result of single- and double-stranded DNA breaks that are unrepaired or incorrectly repaired by repair mechanisms, especially homologous recombination. Sister chromatid exchanges take place just after replication and in subsequent stages of the cell cycle, when chromatids are held together in a cohesive complex that ensures the proximity necessary for exchange between homologous DNA sequences [7]. SCE generation may also be caused by non-homologous recombination [8,9]. This system is fast but not accurate, and therefore any errors in repair are visible in the form of exchanges. These errors disturb the integrity of the genetic material of the cell and the organism [10]. The SCE test is a very sensitive technique and should be carried out with great precision and care. The test result provides information on an animal’s health condition. It can also be used as a tool to assess the genomic stability of a given breed or species.

Fragile sites on chromosomes are another type of chromosomal instability. These are chromosome regions that are particularly susceptible to damage. They can be observed at the cytogenetic level in the form of breaks, gaps or constrictions on chromosomes caused by damage to multiple nucleotide repeats in DNA [11,12]. These repeated nucleotide sequences are able to form specific secondary structures blocking replication forks, resulting in delayed replication [13,14]. These sites are difficult to replicate, and structural rearrangements of the chromosome often occur [11,15]. They are particularly associated with sister chromatid exchange, and are even referred to as ‘hotspots’ of increased frequency of sister chromatid exchange [12,13]. Unreplicated DNA fragments are susceptible to breakage due to the tensions generated by the forces of the karyokinetic spindle pulling sister chromatids to opposite poles. Alternatively, following cell division they can become single-stranded regions, resulting in damage referred to as gaps. These gaps, can be transformed into double-stranded DNA breaks as a result of nuclease activity [16]. Unrepaired damage is generated as FS.

Cytogenetic testing of cattle usually involves analysis of chromosomes treated with Giemsa stain. Differential staining is performed only in justified cases, e.g. in cytogenetic control of bulls used for insemination. Karyotype analysis to detect chromosomal instability is used to identify individuals with genetic defects affecting fertility, reproduction and health. Cytogenetic tests are a particularly valuable tool in assessing the health of animals, but also in evaluating the genome stability of breeds and species. They enable simple, rapid and inexpensive assessment of animals without generating economic losses in breeding [3]. The normal karyotype of domestic cattle is composed of 60 chromosomes. Autosomal chromosomes are acrocentric. The X chromosome is submetacentric, and the Y chromosome metacentric [17]. The acrocentricity of most chromosomes creates major difficulties in their identification and thus in evaluation of the karyotype [18]. For cattle, a standard for Ag-NOR, C, G, Q, and R banding patterns has been established [18–20]. Livestock animals, especially cattle, are attractive models in biomedical research [21]. The cattle karyotype contains about 22000 genes. It can be an important source of information for understanding the evolution of mammals [22]. The Bovidae family exhibits genetic conservatism, which enables comparative studies of the genomes of different animal species. Genome maps are developed based on homology of chromosomes or chromosome fragments [23].

Poland is one of the leading European countries in terms of cattle breeding. The highly productive Polish Holstein-Friesian breed is the top dairy breed, while Limousine is the leading meat breed [24]. Native breeds account for only a small percentage and are counted among indigenous populations that increase biodiversity: Polish Red, Polish Red-and-White, White-Backed, and Polish Black-and-White [25]. They are characterized by low productivity, which is compensated for by their excellent capacity to adapt to unfavourable environmental conditions. They are distinguished by low nutritional requirements, very good feed conversion, resistance to disease, longevity, fertility, and easy calving [26]. The diversity of cattle breeds raised in Poland prompted us to assess these breeds in terms of chromosomal instability.

The aim of the study was to assess the genome stability of ten breeds of cattle raised in Poland using SCE and FS tests.

## Material and methods

This study was carried out in strict accordance with the recommendations in the Directive 63/2010/EU and the Journal of Laws of the Republic of Poland of 2015 on the protection of animals used for scientific or educational purposes. The study was approved by the Polish Local Ethical Commission, Warsaw, Poland (Number: 51/2015) and by the Polish Laboratory Animal Science Association (Number: 3235/ 2015; 4466/ 2017).

The research material was the peripheral blood of ten cattle breeds reared in Poland: Polish Red, Polish Red-and-White, White-Backed, Polish Black-and-White, Polish Holstein-Friesian, Simmental, Montbéliarde, Jersey, Limousine and Danish Red. The first four breeds are covered by a genetic resources conservation programme in Poland. Only material from females was analysed. Blood from the analysed cow breeds was collected from December to March, when the cows were kept indoors. All cows were from one region of Poland (Podlasie). Blood was collected from ten cows of each breed. The age of the animals varied from 2.5 to 8 years. The cows were divided into two age groups. Group 1 consisted of young cows (up to 5 years), and the Group 2 comprised older cows (over 5 years). There were five animals of each breed in each age group.

The peripheral blood was subjected to two different in vitro culture processes depending on how it would be used to identify different types of chromosome damage: sister chromatid exchanges and the identification of fragile sites.

### Cell culture

Mitotic chromosomes were cultured in vitro for 72 hours at 38.5°C. The culture mixture included peripheral blood lymphocytes with serum, RPMI 1640 medium, LF-7 bean plant extract, and antibiotics (penicillin and streptomycin). At 69 h of the culture colchicine was added. At 24 h BrdU (5-bromodeoxyuridine) was added to the cultures intended for SCE assays, and at 65 h BrdU was added to the cultures intended for the FS test. As a hypotonic solution we used 0.65% KCl (potassium chloride). The cells were fixed with Carnoy fixative (3:1 methanol-acetic acid).

### Sister chromatid exchange

The FPG technique [27] was used to detect sister chromatid exchanges in the following steps: digestion with 0.01% RNase, incubation in a solution of 0.5 × SSC with Hoechst solution, UV irradiation twice, overnight incubation at 4°C, incubation at 58°C, and Giemsa staining. Stained sister chromatid exchanges were counted in 20 metaphases from each individual.

### Fragile sites

Microscope slides were stained according to Perry and Wolff [28] in the following steps: incubation in Hoechst 33258 solution, UV irradiation, incubation in 2xSSC, and Giemsa staining. Twenty metaphases were examined from each individual. Chromatid breaks, chromatid gaps and chromosome breaks were identified.

### Statistical analysis

The results were subjected to statistical analysis using STATISTICA 12.5 MR1 PL software. The results were presented in the form of means and standard deviation in breed and age groups. The influence of breed and age on the incidence of chromosomal instabilities (SCE and FS) was determined by two-way analysis of variance using the following model:

$$y_{ijl}=m_{}+a_{i}+b_{j}+{ab}_{ij}+e_{ijl}$$

where: y_ijl_−value of characteristic (mean number of SCEs/cell or mean number of FSs/cell, m–mean for population, a_i_−effect of i^th^ level of factor A (breed), b_j_−effect of j^th^ level of factor B (age), ab_ij_−effect of interaction between factors A and B, e_ijl_−sampling error.

The Tukey test (P <0.05) was used to assess the significance of differences between means for a given type of instability within factors (breed and age). The correlations between the two forms of instability (SCE and FS) in the cattle population were calculated at P <0.05.

## Results

The study evaluated the chromosome stability of ten breeds of cattle raised in Poland. Cytogenetic tests were used to identify instabilities in the cows, i.e. the frequency of sister chromatid exchanges and of fragile sites on chromosomes. Over 4000 metaphases and 240000 chromosomes were analysed.

**Fig 1:**
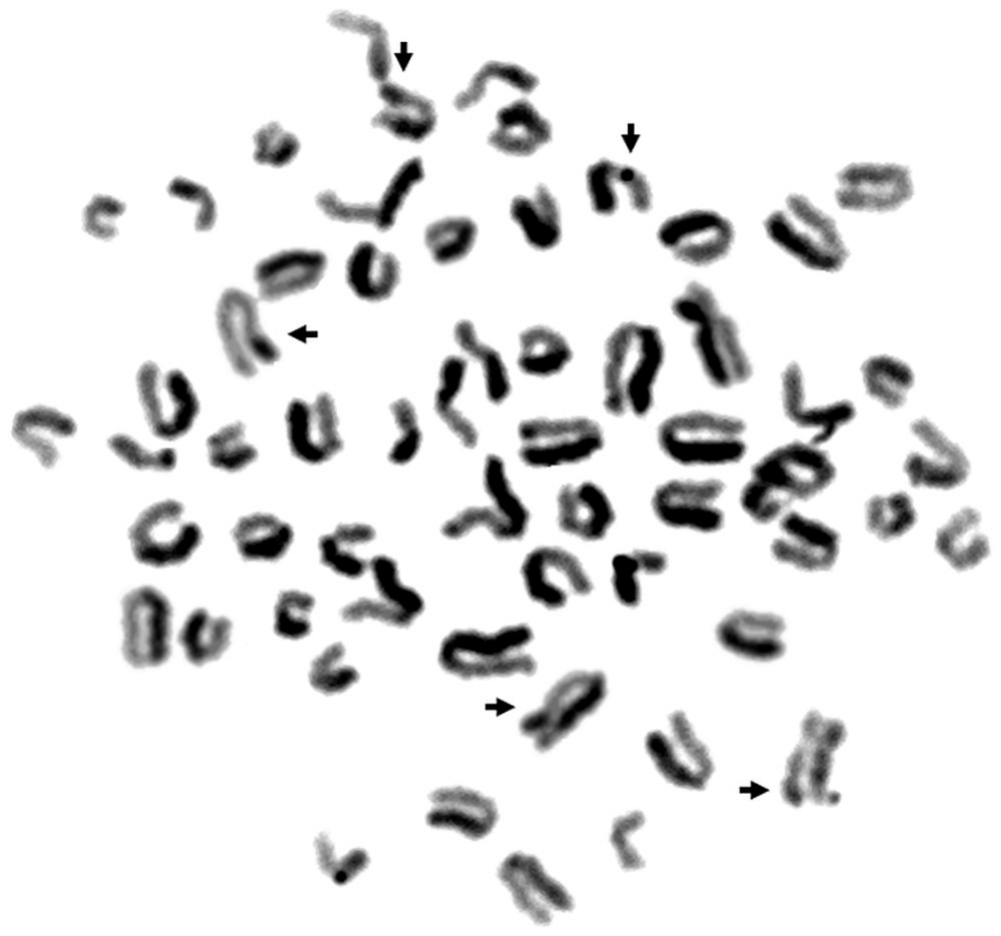
Metaphase plate of cattle chromosomes with identified SCE.

We observed a total of 10163 errors in the genetic material that had arisen during the replication process and were visualized as SCEs. The average frequency of exchanges in the cells was 5.08 ± 1.31. The most SCEs were observed in Polish Holstein-Friesian cows, and the fewest in the Polish Red breed. Statistically significant differences were noted between the Polish Red breed and the breeds Polish Red-and-White, Polish Holstein-Friesian, Simmental, Jersey, and Limousine. Differences were found between White-Backed cows and the breeds Polish Holstein-Friesian, Simmental, Jersey, and Limousine, as well as between Polish Black-and-White cows and the breeds Polish Holstein-Friesian, Simmental and Jersey, and also between Jersey cows and the breeds Polish Red, Polish Black-and-White, Polish Holstein-Friesian and Montbéliarde (Table 1). The assessment of the incidence of SCEs in the cattle breeds showed that the breeds with the least damage to the genetic material, e.g. Polish Red, White-Backed and Montbéliarde, had the most stable genomes. Fig 1 presents an image of a metaphase plate with sister chromatid exchanges registered on cow chromosomes. Analysis of the frequency of exchanges in young and old cows reveals a higher incidence of chromosome damage in older cows than in young ones (5.34 ± 1.24, 4.84 ± 1.25). The differences were statistically significant (P = 0.000). Differences in the incidence of SCEs in different age groups were observed in each breed (Table 2). A significantly lower level of instability was observed in subjects from group 1 than in individuals from group 2. Age was found to significantly affect the level of damage observed. There was no interaction between the factors analysed (age and breed).

**Table 1:** Distribution of SCE/ Cell and FS/ Cell in each breed of cattle.

Breed	SCE/ Cell		FS/ Cell	
	Σ	Mean ± SD	Σ	Mean ± SD
**Polish Red**	860	4.30 ± 1.28^e^	530	2.65 ± 0.86^a^
**Polish Red-and-White**	1001	5.01 ± 1.13^bcd^	640	3.20 ± 0.85^b^
**White-Backed**	920	4.60 ± 1.24^ed^	491	2.46 ± 0.80^a^
**Polish Black-and-White**	976	4.88 ± 1.33^cde^	705	3.53 ± 0.81^cd^
**Simmental**	1046	5.23 ± 1.12^bc^	737	3.69 ± 0.93^de^
**Montbéliarde**	950	4.75 ± 1.27^cde^	674	3.37 ± 1.07^bc^
**Jersey**	1118	5.59 ± 1.00^b^	785	3.93 ± 1.15^ef^
**Polish Holstein-Friesian**	1280	6.40 ± 1.43^a^	848	4.24 ± 1.33^g^
**Limousine**	1056	5.28 ± 0.88^bc^	817	4.09 ± 1.10^fg^
**Danish Red**	956	4.78 ± 1.04^cde^	678	3.39 ± 0.99^bcd^

Values with different lowercase letters differ significantly at P≤0.05.

**Table 2:** Distribution of SCE and FS in the chromosomes of the cow breeds in each age group.

Breed	SCE/ Cell			FS/ Cell		
	1	2	P value	1	2	P value
	Mean ± SD			Mean ± SD		
**Polish Red**	4.05 ± 1.34	4.55 ± 1.18	0.010	2.36 ± 0.81	2.94 ± 0.82	0.000
**Polish Red-and-White**	4.80 ± 1.11	5.21 ± 1.12	0.010	2.90 ± 0.76	3.50 ± 0.85	0.000
**White-Backed**	4.32 ± 1.12	4.88 ± 1.24	0.006	2.16 ± 0.81	2.75 ± 0.67	0.000
**Polish Black-and-White**	4.49 ± 1.24	5.27 ± 1.30	0.000	3.27 ± 0.78	3.78 ± 0.77	0.000
**Simmental**	5.15 ± 0.90	5.31 ± 1.02	0.004	3.37 ± 0.95	4.00 ± 0.80	0.000
**Montbéliarde**	4.47 ± 1.23	5.03 ± 1.24	0.000	2.94 ± 0.94	3.80 ± 1.02	0.000
**Jersey**	5.38 ± 0.94	5.80 ± 1.02	0.001	3.60 ± 1.07	4.25 ± 1.26	0.000
**Polish Holstein-Friesian**	5.96 ± 1.17	6.84 ± 1.52	0.000	3.77 ± 1.36	4.71 ± 1.11	0.000
**Limousine**	5.11 ± 0.96	5.45 ± 0.75	0.002	3.65 ± 1.07	4.52 ± 0.93	0.000
**Danish Red**	4.60 ± 1.05	4.96 ± 0.95	0.005	2.99 ± 0.86	3.79 ± 0.95	0.000

**Fig 2:**
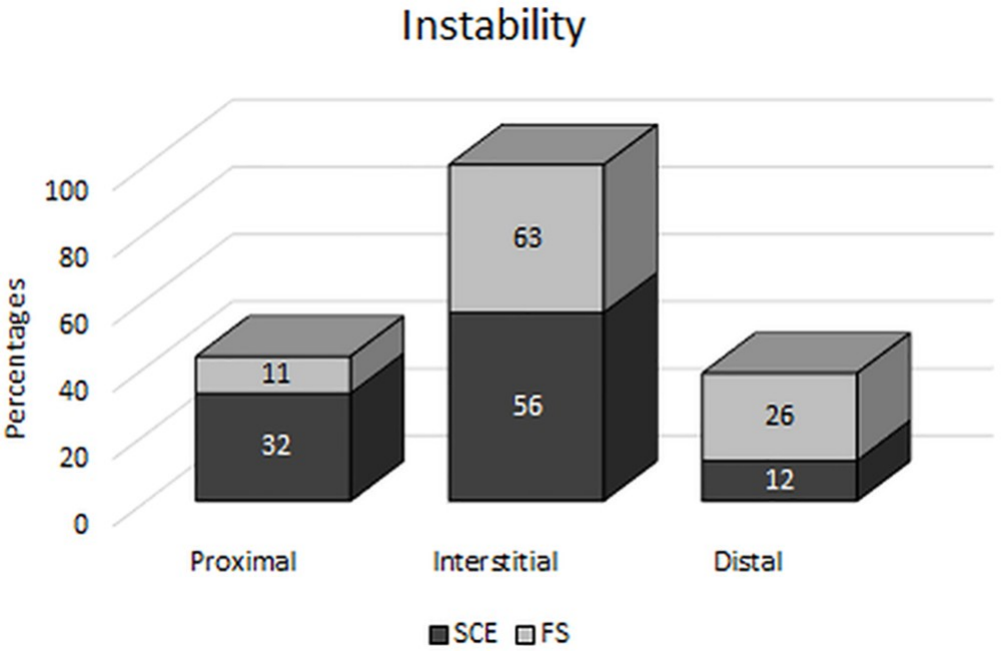
Percentage distribution of SCE/ Cell and FS/ Cell in different parts of the chromosome.

The sister chromatid exchanges occurred in various parts of the chromosome: in the proximal, interstitial and distal parts. The most exchanges were observed in the interstitial part of the chromosome, and the fewest in the distal part (Fig 2).

**Fig 3:**
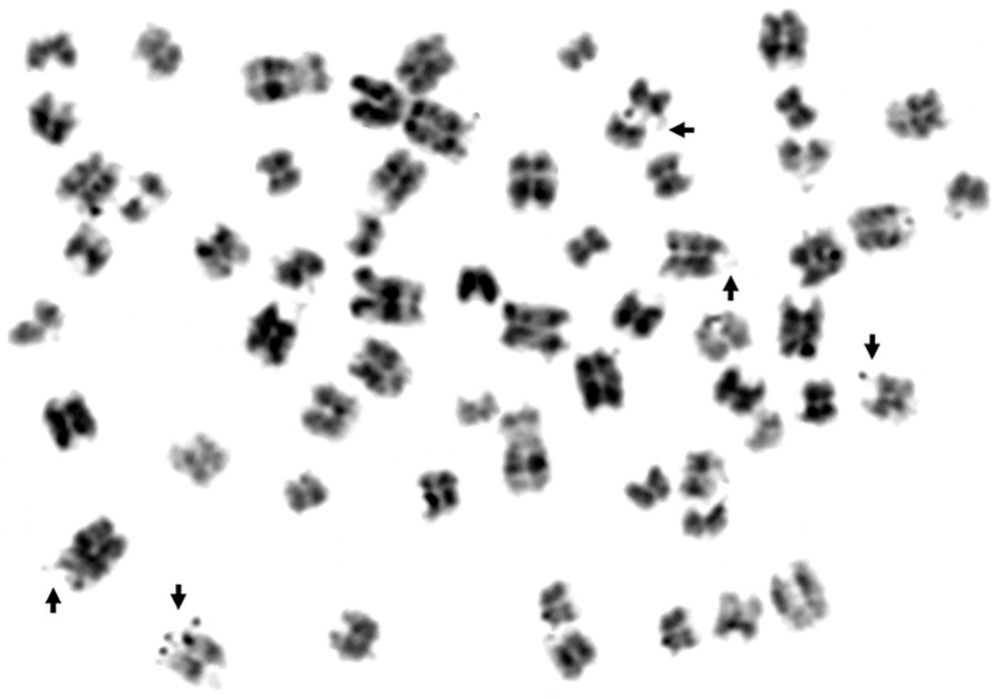
Metaphase plate of cattle chromosomes with identified FS.

The study also assessed damage to genetic material in the form of fragile sites on chromosomes (Fig 3). These instabilities were identified in the form of DNA breaks in one strand or in both, contributing to the formation of chromatid gaps and breaks and even to deletion. In total, 6905 fragile sites were found on the chromosomes, with an average frequency of 3.45 ± 0.94. Damage was most common in the interstitial part of the chromosome, and the least common in the proximal part (Fig 2).

The most FSs were observed in Polish Holstein-Friesian cows and the fewest in Polish Red. There were no statistically significant differences between Polish Red and White-Backed; Polish Black-and-White and Simmental; Polish Black-and-White and Jersey; Polish Red-and-White and Montbéliarde; or Jersey and Limousine (Table 1). In addition, age was found to influence the level of damage in each of the breeds analysed. The differences observed in the incidence of fragile sites on chromosomes were statistically significant (P = 0.000). The cows in group 1 had a lower frequency of damage than the animals in group 2 (3.10 ± 0.88 and 3.80 ± 0.92, respectively; Table 2). No interaction between the age and breed factors was found. The White-Backed breed was shown to be the most genetically resistant to the negative effects of genotoxic, mutagenic and carcinogenic factors, having the fewest fragile sites. It was determined to have the most stable genome among the analysed cattle breeds. Animals with a large number of fragile sites were identified as less genetically resistant, and the Polish Holstein-Friesian breed was considered to have a less stable genome than the other cattle breeds.

The correlation coefficient between the tests used to identify sister chromatid exchanges and fragile sites was r = 0.84. A correlation was found between the techniques for identifying sister chromatid exchanges and fragile sites.

## Discussion

Cattle are the most widespread livestock animal and the main source of food of animal origin. Over the years there has been a huge increase in cattle production for both milk and meat. This has been possible mainly due to intensive assessment of utility and breeding value as well as selection for milk or meat traits. At the same time, breeders’ exclusive focus on increasing productivity has entailed neglect of the functional features of cattle, leading to a decline in health, reproductive performance and biodiversity, as well as the emergence of defects and genetic diseases. Effective assessment and detection of abnormalities is extremely important, as they pose the greatest risk of economic losses for the breeder.

Cytogenetic karyotype analysis can be used for genetic evaluation of species, and thus can be a valuable tool for improving livestock [29]. It enables analysis of genomic stability. Cytogenetic tests can be used to assess the negative impact of mutagenic, genotoxic and carcinogenic factors on the animal. Examination of chromosomal instability includes many different cytogenetic tests, including very sensitive tests for sister chromatid exchanges and the identification of fragile sites on chromosomes. Sister chromatid exchanges on chromosomes can be visualized using cytogenetic methods involving staining of sites on chromosomes in which errors have occurred during replication [3,6,30].

Due to the universality of the SCE test, it has been used to assess the genome stability of various animal species, and the results of these studies indicate species conservatism for the incidence of SCE. Research conducted on chickens by Arias [31] and Wójcik et al. [32,33] provides valuable information on the incidence of SCEs depending on the age of the birds. In embryos, the frequency of SCEs was very low, at 1.2, while in laying hens it was 7.8. These differences are due to the age of the animals. The older the animals, the more SCEs are observed. There have been few studies on the influence of age on the incidence of SCEs. Research on horses by Wójcik et al. [34], Wójcik and Smalec [35] and Wnuk et al. [36] has shown a higher frequency of SCEs in old horses than in young ones. The same relationship was observed by Peretti et al. [37] in their analysis of the level of instability in pigs. In studies on humans, a positive correlation has also been observed between age and the incidence of SCEs [38–40]. This relationship was also confirmed in the present study, as differences in the incidence of SCEs were found in the two age groups. In each breed, the incidence of SCE was lower in the first group, comprising young cows, than in the other group. The relationship observed between age and the level of damage is associated in part with ineffective repair mechanisms of damage to genetic material. Therefore, SCE is considered a measure of repairs of damaged DNA in the body. Unfortunately, the effectiveness of repair mechanisms decreases with age, and they generate more errors. The effectiveness of checkpoints tasked with detecting damage and directing it onto the repair pathway declines as well. The inefficiency of these mechanisms increases the body’s susceptibility to the occurrence and accumulation of instabilities. The cell becomes more sensitive to mutagenic factors [41]. The results confirm that ageing increases the level of DNA damage, which is caused in part by a decline in the efficiency of control and repair mechanisms. It can be concluded that as animals age, their use for reproduction should be limited, due to the increased risk of genetic defects passed on to the next generation. Researchers have also observed different frequencies of sister chromatid exchanges within species, despite high conservatism. This is influenced by the breed of the animals. Many comparative studies on various livestock species have shown that breed is the factor that significantly affects the incidence of SCE [34,42–45]. Iannuzzi et al. [44], in a study on various breeds of cattle, i.e., Podolian, Friesian and Romagna found different frequencies of SCE in the breeds (7.9, 7.1, 7.3). Different SCE rates have been obtained by Leibenguth and Thiel [46] in German Black-and-White and Red-and-White cattle (8.3), by Vijh et al. [47] in Sahiwal cattle (3.2), by Rangel-Figueiredo et al. [48] in Marones cattle (6.6), by Ciotola et al. [42] in Agerolese cattle (5.4), and by Azimi Dezfouli [49] in Holstein-Friesian cattle raised in Iran (6.8). The diverse results obtained by researchers are indicative of the influence of the breed, but may also be due to the use of animals of different ages in the research. In the present study analysing ten breeds of cattle, differences were also found in the incidence of SCEs, which ranged from 4.3 to 6.4. The lowest incidence of SCE was found in the Polish Red breed, followed by White-Backed, Montbéliarde, Danish Red, Polish Black-and-White, Polish Red-and-White, Simmental, Limousine, Jersey and Polish Holstein-Friesian. The low SCE rate observed in indigenous breeds is associated with a more stable genome, resistant to various types of damage to genetic material. Similar results have been obtained by Wójcik et al. [32,34], who analysed various breeds of horses, including indigenous ones, and chickens, including one indigenous breed. Four of the ten cattle breeds analysed are covered by a genetic resources conservation programme, so great emphasis has been placed on keeping the gene pool at a constant level. The Montbéliarde breed is also an old breed, originating in France, and has characteristics of local breeds. Production breeds, on the other hand, are still continually being improved through strict selection, so that their gene pool is always changing. An additional factor is the influence of breeding with imported cattle characterized by very good dairy or meat traits. The higher average SCE rates observed in the other cow breeds may be due in part to intensive breeding work on these breeds.

The frequency of sister chromatid exchanges is a complex multifactorial trait. According to Wójcik and Smalec [35], in addition to the factors described above, as well as varied selection pressure, the SCE rate is also influenced by the animal’s living environment. Exogenous compounds that enter the body together with food, drink and air adversely affect genome stability. Cattle breeds kept indoors are less exposed to the negative effects of environmental pollution than those that graze on pastures [45]. These animals live far from urban areas, in unpolluted areas of Poland. Thus the cows had not been exposed to harmful environmental factors and for this reason the level of damage was not high in comparison with breeds described by other researchers. According to Azimi Dezfouli [49], the average SCE rate for healthy cattle ranges from 5 to 14 exchanges. Any significantly large deviations from the standard may indicate pathological changes that adversely affect the state of the genome [33]. A high incidence of SCE indicates reduced genomic stability, which in turn is associated with an increased likelihood of mutations [37]. In our own research, the cattle population had an average frequency of sister chromatid exchanges of 5.1. This is much lower than the average values reported for the species by other researchers.

Many scientists investigating sister chromatid exchanges claim that the sites where exchanges occur are not accidental and show species-specific variation. The location of SCEs in the chromosome is likely to be associated with different arrangements of heterochromatin in different species [32,50]. According to Latt [51] and Iannuzzi et al. [52], SCEs are generated in the G-bands and in the areas between the bands. Arias [31], Wójcik et al. [34] and Wójcik and Smalec [35,43] observed the most SCEs in the interstitial part of the chromosomes, in sites where heterochromatin borders with euchromatin, and many fewer in the proximal part. In the present study as well, most of the SCEs observed in the cattle population were in the interstitial part of the chromosomes, followed by the proximal part, while the fewest were found in the distal part of the chromosome. According to many researchers, exchanges take place less frequently in the terminal part of chromosomes [32,53]. On the other hand, Kuchta-Gładysz et al. [50,54] have reported a very high level of damage in the distal part of the chromosome. Damage in this part of the chromosome is caused by oxidative stress, which results in single-strand DNA breakage in the telomeres. Blagoev et al. [55] even stated that telomeres are SCE hotspots.

Analysis of SCEs under a microscope is quite easy; it involves identifying discontinuities in chromatid staining in the chromosome. Identification of instabilities, referred to as fragile sites, is much more difficult and labour-intensive [13]. Both methods are used to assess and compare the genome stability of species as well as breeds of animals, including livestock [34,44,56]. Fragile sites are specific areas on chromosomes that exhibit fragility under certain conditions. Their location in the structure of metaphase chromosomes is not accidental [53]. Fragile sites are areas with a tendency towards changes in the DNA sequence, which makes them unstable regions in the genome. This instability can be observed using fairly simple cytogenetic methods to detect damage to chromosomal structure, involving lymphocyte cultures in specific in vitro conditions in the presence of nitrogen base analogues [57]. FS-inducing agents include mutagenic and clastogenic chemicals [58]. Fragile sites must be induced with chemical compounds because the spontaneous expression of fragile sites common on metaphase plates is very low (less than 5%), which makes them very difficult to detect [59]. Fragile sites are a natural element in the chromosome structure of all species and show genetic conservatism in the evolution of mammals [60]. In cattle, Di Berardino et al. [61] were the first to attempt to identify FS. The authors determined the frequency of fragile sites to be 3% (average frequency FS 0.21/cell). Peretti et al. [62] have observed chromosome damage with a frequency of 0.02 to 2.8/cell in Italian cattle. A low frequency of damage on bovine chromosomes has also been observed by Di Meo et al. [63] in a Piedmontese x Valdostana crossbreed (0.1–0.7) and in the Valdostana breed (0.1–1.4). Danielak-Czech and Słota [64] reported spontaneous FS in Polish Black-and-White cattle at a level of 3.0/cell (without BrdU), while in the case of induction with BrdU and 5-AZA at 50 ug mL^-1^, they observed an increase in the frequency of damage (39.8 and 36.2, respectively). In subsequent research on cattle, Danielak-Czech et al. [65] reported chromosome fragility at a level of 27.2% in the case of induction with APC and 9.95% for induction with folates. Rodriguez et al. [59], in their analysis of FS in Holstein-Friesian cattle, found a level of damage ranging from 2.5% to 92.3%. In our own research, the frequency of fragile sites in the total population of tested cattle was 3.45 FS/cell. We observed a slightly higher frequency of FS in Polish Black-and-White cattle (3.53) following induction with BrdU in comparison to the frequency obtained by Danielak-Czech et al. [65] without an inducer (3.0), and a much lower frequency than they obtained using 50 ug mL^-1^ BrdU (39.8/cell). In our study, different FS frequencies were observed in each of the breeds. The fewest FS were observed in White-Backed cattle, followed by Polish Red, Polish Red-and-White, Montbéliarde, Danish Red, Polish Black-and-White, Simmental, Jersey, Limousine and Polish Holstein-Friesian. As in the case of SCE, the least damage was observed in native breeds and the most in Holstein-Friesian. Local breeds are primitive breeds, with lower use value, but they are resistant to harsh environmental conditions and are characterized by good health and hardiness. Increased damage has been observed in breeds subjected to selection pressure. The results of our research show that the breed significantly influences the frequency of fragile sites in chromosomes. This is supported by results obtained by other scientists studying various breeds within species. Different frequencies of FS have been observed in buffalo by Albarella et al. [66], Di Meo et al. [67], Genualdo et al. [68] and Kumar et al. [69]. Different FS values have also been observed in various breeds of pigs [64,70,71], sheep [64,72–74], chickens, and quails [32,75]. The results obtained by these researchers indicate that breed has a major influence on the incidence of FS. Wójcik et al. [75], in a study on quail, evaluated pure breeds (Japanese quail and Pharaoh Coturnix) as well as crosses of these breeds in the F1 and F2 generations. The authors observed the most chromosome damage in the parent generation, slightly less in the F1 generation (crossbreds) and the least in the F2 generation. They showed that the FS identification test is an excellent cytogenetic tool for assessing genome stability and use as a biomarker, and the results of the genomic stability assessment indicate that crossbreeding has a positive effect. The low FS rates in the animals tested in our study also suggest that native Polish cattle breeds have a very stable genome and can be used to improve not only the genome stability of other cattle breeds, but also their functional and health traits. FSs, unfortunately, have a destructive effect on the functioning of many cellular mechanisms. An increased FS frequency negatively affects animal health, causing reproductive problems, various deformities, chondrodystrophy, cancer and other diseases [12,13,64,76–80]. In our study, differences in the incidence of FS in young and old individuals were found for each breed of cows. A higher frequency of FS was observed in old animals than in the group of young animals. There are very few published studies dealing with this question. Ali et al. [74] observed differences in the level of FS between young and old sheep, but they were not statistically significant. A large increase in the expression of fragile sites (3–4 fold) has been found in studies on chromosomal instability in humans. The ageing process can be presumed to be associated with irreversible changes in the genome, which become strengthened and perpetuated during cell division. This increases susceptibility to fragility induced by various factors. Unfortunately, as in the case of sister chromatid exchanges, this kind of instability also results from increased DNA methylation in the cells of old individuals, which is conducive to DNA damage [43]. Schoket [81] and Srám et al. [82] also found that chromosomal fragility increases with age. The authors suggest that this is due to longer exposure of genes to environmental factors. The older the individual, the longer it has been exposed to the negative effects of mutagens.

Our study also investigated the location of fragile sites on the chromosomes of the cattle. The vast majority of FS were located in the interstitial part of the chromosomes, followed by the terminal part, while the fewest fragile sites were located in the proximal part. Danielak-Czech and Słota [64] and Rodriguez et al. [59] made similar observations. They identified fragile sites on cattle chromosomes on the boundary of positive and negative R-bands. According to Le Beau et al. [83], the appearance of FS in R-bands can be explained by late or delayed replication of the fragile sites, which is consistent with the nature of negative R-bands, which are replicated in the late S phase. Ruiz-Herrera et al. [60] found that bands with fragile sites are richer in tandem sequences. Zlotorynski et al. [11], Glover [12], Durkin and Glover [13] and Nicolae et al. [84] have concluded that fragile sites are ‘hotspots’ of sister chromatid exchanges. This is supported by our research, because we found an increased rate of damage, both in the form of SCE and FS, in the interstitial part of the chromosomes of the cattle karyotypes analysed for chromosomal instability.

## Conclusions

The analysis of chromosomal instability in cattle using the SCE and FS tests provided a more complete picture for the assessment of the genetic stability of these individuals. These tests also make it possible to assess the genomic stability of individual breeds, providing valuable information to be used in efforts to preserve biodiversity and to improve animals. Moreover, both the sister chromatid exchange test and the fragile site identification test can be used as bioindicators to identify less healthy animals, which have an increased level of damage in their genetic material. Rapid examination of individuals with genetic disorders allows them to be eliminated from breeding early on. This is particularly important for the breeder, in terms of both economics and breeding. The analysis of the prevalence of instability in various breeds, including indigenous breeds, has shown that native breeds have a more stable genome. These breeds can be crossed with other breeds of cattle to improve their genetic stability as well as their functional traits.
